# The Application of Commercial Surface Acoustic Wave Radio Communication Filters as Transducers for DMMP Sensors

**DOI:** 10.3390/s24134299

**Published:** 2024-07-02

**Authors:** Michał Grabka, Krzysztof Jasek, Mateusz Pasternak, Zygfryd Witkiewicz

**Affiliations:** 1 Faculty of Advanced Technologies and Chemistry, Military University of Technology, 00-908 Warsaw, Poland; krzysztof.jasek@wat.edu.pl (K.J.); zygfryd.witkiewicz@wat.edu.pl (Z.W.); 2 Faculty of Electronics, Military University of Technology, 00-908 Warsaw, Poland; mateusz.pasternak@wat.edu.pl

**Keywords:** surface acoustic wave (SAW), chemical sensor, radio communication SAW, nerve CWA detection

## Abstract

In the present study, we used two popular radio communication SAW resonators as a base for gas sensors and tested their performance. Taking into account issues related to sensor sensitivity, the possibility of applying a sensor layer, the availability of devices, and other related issues, we selected two popular single-port resonators with center frequencies of 315 and 433 MHz (models R315 and R433, respectively) for testing purposes. Both resonators were equipped with a sensitive film of hexafluoroisopropanol-substituted polydimethylsiloxane, a material that selectively absorbs molecules with a high ability to form basic hydrogen bonds. Fabricated sensors were used to detect trace amounts of dimethyl methylphosphonate (DMMP) vapor, which has often been used in similar studies as a nerve chemical warfare agent simulant. Sensors using both devices loaded with sensor layers of an optimal thickness rapidly reacted to a gas containing DMMP at a concentration of 3 mg/m^3^, generating a stable analytical signal ranging from several to several dozen kilohertz. In the case of R433, the frequency signal was 20.5 kHz at 1 min from the beginning of exposure to DMMP. The obtained results showed that the used transducers exhibited good performance as a base for gas sensors. Finally, their suitability for sensing applications was confirmed by a comparison with the results obtained in previous similar studies.

## 1. Introduction

As is commonly known, various kinds of surface acoustic waves (SAWs) are highly sensitive to interactions with external factors. Even slight variations in environmental parameters result in significant changes in the amplitude, velocity, or phase of such waves. This property is a disadvantage for SAW signal-processing devices, like filters or resonators, but it is very desirable for the construction of sensors The popularity of these sensors stems from the fact that wave parameter changes are relatively easy to measure. A number of qualitative and quantitative analyses of the operational principles of sensors have been published in the literature [1,2,3]. Based on this theoretical background, many innovative SAW sensor constructions are manufactured today, forming a large family of acoustoelectronic transducers [4]. Different kinds of chemical sensors also belong to this family.

Regardless of the sensing mechanisms used, all of these devices have to employ some type of acoustoelectronic system that generates and detects SAWs. Commonly, two interdigital transducers (IDTs) working on a piezoelectric substrate are used for this purpose. The first generates a SAW based on the converse piezoelectric effect; the second detects it, based on the direct piezoelectric effect. A set of such IDTs placed one after the other and sufficiently close to each other forms a SAW delay line, one of the simplest acoustoelectronic devices. It is also possible to use only one IDT that carries out the twin roles of generation and detection. In this case, it has to work between two SAW reflectors. Such a configuration has Fabry–Pérot resonator properties and is called a one-port SAW resonator. If the IDT and reflectors form a continuous array with a constant period, the device is referred to as synchronous. Of course, it is possible to utilize multiport devices with more (commonly two) IDTs between the reflectors. There are a number of possibilities for acoustic reflector constructions, but planar Bragg gratings made of periodic surface metal strips are those most often applied.

As already noted, the construction of any type of SAW sensor requires the use of an acoustoelectronic device as a base transducer. Because the technology of manufacturing such devices is not widely accessible and usually involves considerable costs, the small-scale production of SAW devices, especially those dedicated to sensor experiments, is uneconomic. For this reason, sensor constructors and researchers often use common commercial products like delay lines or resonators [5]. Today, a large number of manufacturers offer many low-cost SAW devices of such kind. To gain access to the surface of a commercial product, it is enough to carefully cut off the cap of its factory casing. After some additional treatment, like chemical or plasma cleaning, the surface is then ready for a chemosensitive layer to be deposited [6]. Such a solution may not be very sophisticated, but it is effective and involves minimal cost. Moreover, the consistency of commercially manufactured products guarantees good repeatability in experiments in which they are used. A review of the literature concerning the construction of different SAW sensors shows that the device most often chosen by researchers for this purpose is a one- or two-port resonator [6,7,8,9,10]. Unfortunately, some of these devices are no longer produced, so it is impossible for some experiments to be repeated. In the selection of a suitable device, researchers typically consider the type of device, the possibility of removing its casing, and the value of the center frequency, which determines the achievable sensitivity. Attention is also paid, though less frequently, to the commercial availability of the devices. As a result of these considerations, the most widely used devices employed are 315 and 433 MHz SAW resonators, which are frequently used in remote-control transmitters in Europe [11,12,13].

The proper selection of a suitable SAW device for gas detection experiments determines, to a large extent, the properties of the final manufactured sensor. A number of important features must be taken into consideration, including the type of substrate, the surface waves, the device itself, and the device’s geometry. The substrate determines the *Q* factor of the device, its sensitivity, and its thermal stability. Due to these key parameters, ST-cut quartz is often chosen [14]. The type of wave is also important from the point of view of sensitivity. The transversal and Love modes seem to be the best choice in this regard. These modes usually offer sensitivity two times higher than Rayleigh modes [15]. Unfortunately, the majority of commercial devices operate using the Rayleigh basic mode. The last factor, geometry, determines the kind of device and its center frequency. Some analyses have shown that it is better to choose an SAW resonator rather than a delay line [16]. As is commonly known, the sensitivity of an SAW sensor depends on its center frequency [4]. Higher frequencies mean that the device has a smaller area and a lower mass change at a constant film thickness, so a compromise must be found between the frequency and the effective area of the device.

An analysis of the acoustoelectronic properties of a larger set of different devices working with frequencies from several to several hundred MHz suggests that the popular synchronous R315 and R433 one-port SAW resonators may be most suitable for experimental purposes. These devices are produced by many manufacturers, and because of their many radiofrequency applications, they most probably will remain in production for a relatively long time. The typical structures of such devices make them suitable for sensor experiments. The surfaces of such resonators are almost entirely covered by a uniform array of metal electrodes. Such a geometry allows for the sensitive layers to be deposited on the entire surface area of the devices, so that homogeneity of loading is maintained. The resonators work with the basic Rayleigh wave mode on ST-cut quartz. The substrate and specific planar geometry allow for a low-frequency temperature coefficient and high *Q* factor. Additionally, both devices are built in housings that are relatively easy to open (HC49, TO 39, or F11).

In this paper, we present the results of our research on gas sensors using two types of commercial SAW filters with fundamental frequencies of 315 and 433 MHz. Polymer sensing layers of various thicknesses were applied to these devices, which enabled the useful load ranges of the resonators to be estimated. The polymer material used was a polydimethylsiloxane copolymer modified with fluoroalcohol substituents with a high ability to sorb hydrogen bond bases, including nerve chemical warfare agents (CWAs) and CWA simulants. Measurements with DMMP (a commonly used nerve CWA simulant) enabled us to estimate the suitability of both devices for detecting compounds of this type.

## 2. Materials and Methods

### 2.1. Materials and Instrumentation

**Figure 1:**
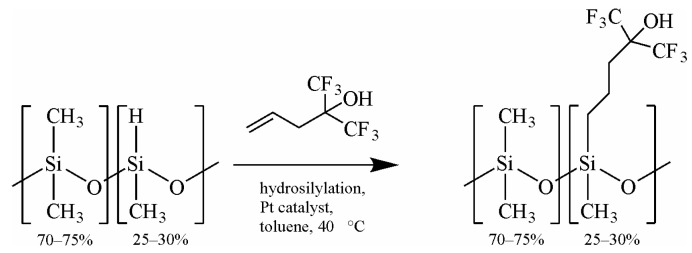
Synthesis of sensor coating material: copolymer 25–30% [3-(1,1,1,3,3,3-hexafluoropropan-2-ol)propyl]methylsiloxane and dimethylsiloxane.

The chemical reagents used for cleaning SAW devices, such as acetone (99.5%, POCH, Gliwice, Poland) and ethanol (99.8%, POCH, Gliwice, Poland), and for sensor testing, dimethyl methylphosphonate-DMMP (97%, Alfa Aesar, Haverhill, MA, USA), were used in the form in which they were supplied. Additionally, THF, which had previously been purified using the benzophenone–sodium still method, was used for the final cleaning of the resonators and the preparation of polymer solutions. For the present study, we used a copolymer of 25–30% [3-(1,1,1,3,3,3-hexafluoropropan-2-ol)propyl]methylsiloxane and dimethylsiloxane, which was synthesized in our laboratory (the structure of the polymer is shown in Figure 1).

This is a highly hydrogen-bonding acidic material that relatively selectively absorbs basic vapor such as nerve CWAs and their simulants. The sorption mechanism is based on the formation of hydrogen bonds between analyte molecules and the polymer material. In this case, the polymer acts as a hydrogen atom donor for bonding (it is the acid of a hydrogen bond), effectively binding molecules with a high ability to act as an electron-pair donor (hydrogen-bond base). Due to the fact that nerve CWAs show high basicity in terms of hydrogen bonds, this mechanism is considered relatively selective and is used for the detection and preconcentration of compounds of this type.

The synthesis of the material involved modifying the chain of a copolymer of dimethylsiloxane and methylhydrosiloxane with a Si-H bond content of 25–30% ((25–30% methylhydrosiloxane) dimethylsiloxane copolymer hydride terminated, ABCR, Karlsruhe, Germany) by introducing 2-allyl-1,1,1,3,3,3-hexafluoroisopropanol substituents (2-allylhexafluoroisopropanol, 98%, ABCR, Karlsruhe, Germany) via a platinum-catalyzed (Karstedt, platinum–divinyltetramethyldisiloxane complex in xylene, 2.1–2.4% Pt, ABCR, Karlsruhe, Germany) hydrosilylation reaction. The progress of the reaction was monitored by infrared spectroscopy. The obtained polymer was in the form of a thick, light-gray oil. Synthesis details are provided in the Supplementary Materials.

Surface preparation of the SAW device prior to first use was carried out using a Q150T ES vacuum coater (Quorum Technologies, Lewes, UK) with a glow discharge insert in an atmosphere of ambient air.

### 2.2. Stand for Testing SAW Sensors

The stand for testing SAW sensors consisted of a gas mixture generation system, a measurement chamber in which the tested SAW sensors were placed (the measurement chamber housed a single sensor at a time), a vector network analyzer, and a PC with dedicated software enabling control of the gas mixture generation system (flows, temperature) as well as reading and acquisition of the sensor signal.

The homemade gas mixture generation system allowed for the generation of a gas stream with a specific flow rate and concentration of DMMP vapor, which was passed through a measurement chamber with a SAW sensor. This system used two mass flow controllers (model F-201C, Bronkhorst, Ruurlo, The Netherlands) and a gravimetrically calibrated diffusion source of analyte vapor. The source was constructed in the form of a small glass vessel (liquid analyte reservoir) ended with a capillary. Under thermostatically controlled conditions, the source in the container was maintained at a temperature of 40 ± 0.1 °C (temperature control was based on the FCR-13A-S PID controller (Shinko, Chikuma, Japan)). The source maintained a constant emission of analyte vapors over time, and this was verified gravimetrically (see Supplementary Materials). To prevent the condensation of analyte vapor, the gas pipes leading the gas mixture to the measurement chamber were also heated. The gas system and chamber were fed by synthetic dry air from a pressurized cylinder. Before starting the measurements, a tightness test of the system was performed, and the analyte content in the generated gas was verified using gas chromatography (static headspace sampling). The obtained results confirmed the correct operation of the system.

The measurements were carried out using a vector network analyzer (model miniVNA-Tiny [17], miniRadioSolutions, Jaworze, Poland) working with a PC with proprietary control software. miniVNA-Tiny is a very compact two-port measurement system, which can be used for transmission and reflection measurements on band filters or resonators. The recommended factory software is available for most popular operational systems and can be easily upgraded via a USB interface. This approach is very simple and cheap and provides good repeatability and frequency resolution.

**Figure 2:**
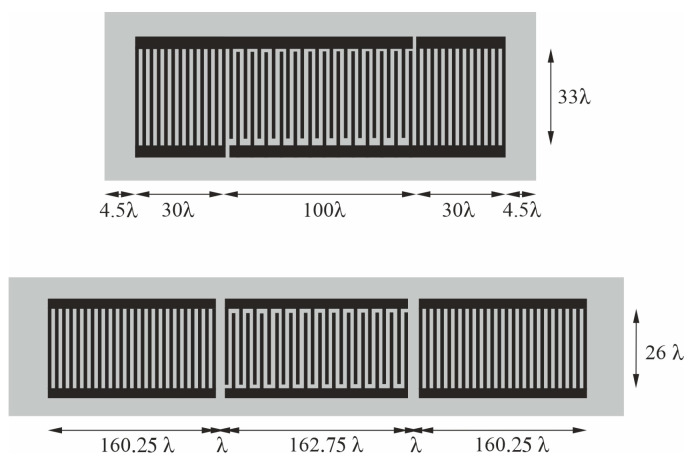
The geometries of one-port synchronous SAW resonators 315 MHz (at the top) and 433 MHz (at the bottom).

Two types of synchronous one-port SAW resonators were used in the present study; these were the 315 and 433 MHz resonators (models R315 F11 [18] and R433 F11 [19], respectively (YXC, Shenzhen, China)). Both of these resonators have an ST-quartz substrate and uniform distribution of electrodes along their entire length. The main difference between the two, apart from their different frequencies, lies in the different way in which their internal reflective structures are produced. In the case of the 315 MHz resonator, the reflective properties of the interdigital transducer, the short array of shorted strips, and the edges of the substrate were exploited; in the case of the 433 MHz resonator, only the reflective properties of the shorted-strip arrays were used. As a result, the 433 MHz resonator was relatively long; in addition, despite its higher frequency, its surface area accessible to sensitive film deposition was approximately 20% larger compared with the 315 MHz resonator. The geometries of both resonators are illustrated in Figure 2.

In both cases, two metal electrodes fit into the one wavelength equal to 10 and 7.293 mm, respectively. The apertures were designed to obtain 50 Ω matching. A summary of the values of typical resonator parameters is shown in Table 1.

The long-term stability of the two transducers was obtained from the catalog notes [18,19]; in both cases, this was typically <10 ppm per year. However, these values apply to resonators tightly closed in their factory housings. No information was available on the long-term stability of a resonator whose substrates are exposed to ambient air and other external conditions and covered with sensor layers, as is the case in gas sensors.

**Figure 3:**
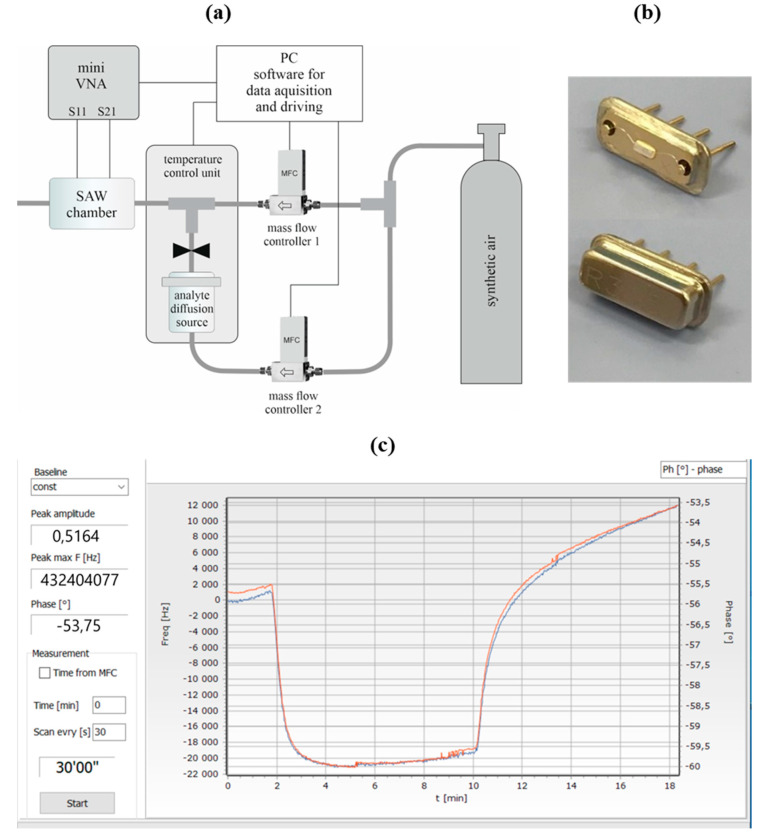
(**a**) Diagram of stand for testing SAW sensors used in this work. (**b**) Photo of R315 resonator both with and without top of the factory casing. (**c**) Print screen of control software (taken during measurements).

A diagram of a stand for testing SAW sensors, a photo of the R315 resonator with the top of its casing removed, and a print screen of the control software are all presented in Figure 3.

## 3. Results and Discussion

### 3.1. Manufacture of Chemical Sensor

To obtain a chemical sensor based on either of the above-mentioned resonators, the upper part of the factory casing must be carefully cut off. As produced by the manufacturer, the resonator itself is clean enough to be suitable for its typical applications; however, for chemical detection, factory surface preparation is often insufficient. Numerous experiments carried out by the authors have shown that laboratory cleaning with high-purity reagents and low-energy ion etching can change the center frequencies of high-frequency resonators by up to hundreds of kHz. This proves that the factory level of cleanliness is not very high.

After the upper part of the factory casing was removed, the SAW device was initially cleaned by successive immersion in acetone, ethanol, and THF. Next, after evaporating the solvent, the device was subjected to glow discharge treatment in ambient air (ion current 25 mA, 10 min, negative mode). Glow discharge treatment was only performed the first time a new resonator was used. After the resonator cleaning process was completed, its frequency characteristics were recorded; these constituted a reference point for determining the thickness of the applied polymer layers. Polymer films were applied to the resonators prepared in this way. The films were applied by immersing the resonators in polymer solutions in THF with various polymer percentages, ranging from 0.1% to approximately 1.5% by weight, and leaving the solvent to evaporate. After the solvent was evaporated, the resonators were heated under an IR lamp for 15 min and further conditioned in a flow of dry air in a measurement chamber at room temperature for another 30 min. After conditioning, the frequency characteristics of the sensor were recorded.

**Figure 4:**
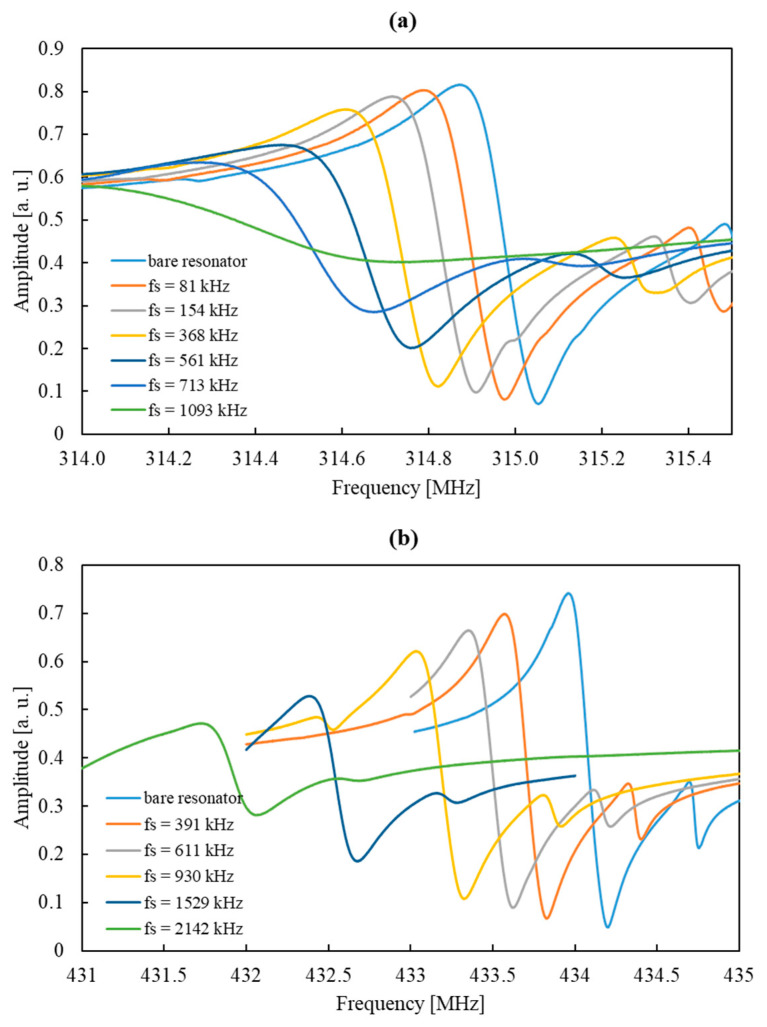
Amplitude characteristics of sensors with varying thickness of polymer coating deposited on the surface of resonators with fundamental frequencies of (**a**) 315 MHz and (**b**) 433 MHz.

It is worth noting that the applied layers could easily be dissolved and washed off using the same sequence of solvents as described above (in this case, with no glow discharge treatment being used), and then, a new layer with different parameters could be applied. The amount of polymer deposited (proportional to the layer thickness) was determined by monitoring the amplitude characteristics of the sensor and calculating the change in the position of the maximum of the resonance peak between the characteristics. The value of this shift, determined after the sensor was manufactured and denoted as *f_s_*, is used later in the present paper to identify individual sensors manufactured using R315 and R433 resonators. The use of solutions with different percentages of polymer allowed for us to obtain sensors with different *f_s_* values. The details of the process of applying polymer layers to the resonators are included in the Supplementary Materials. Figure 4 shows the amplitude characteristics of resonators with resonance frequencies of 315 MHz (R315) and 433 MHz (R433), characterized by different values of *f_s_*.

### 3.2. Measurements with DMMP Vapor

**Figure 5:**
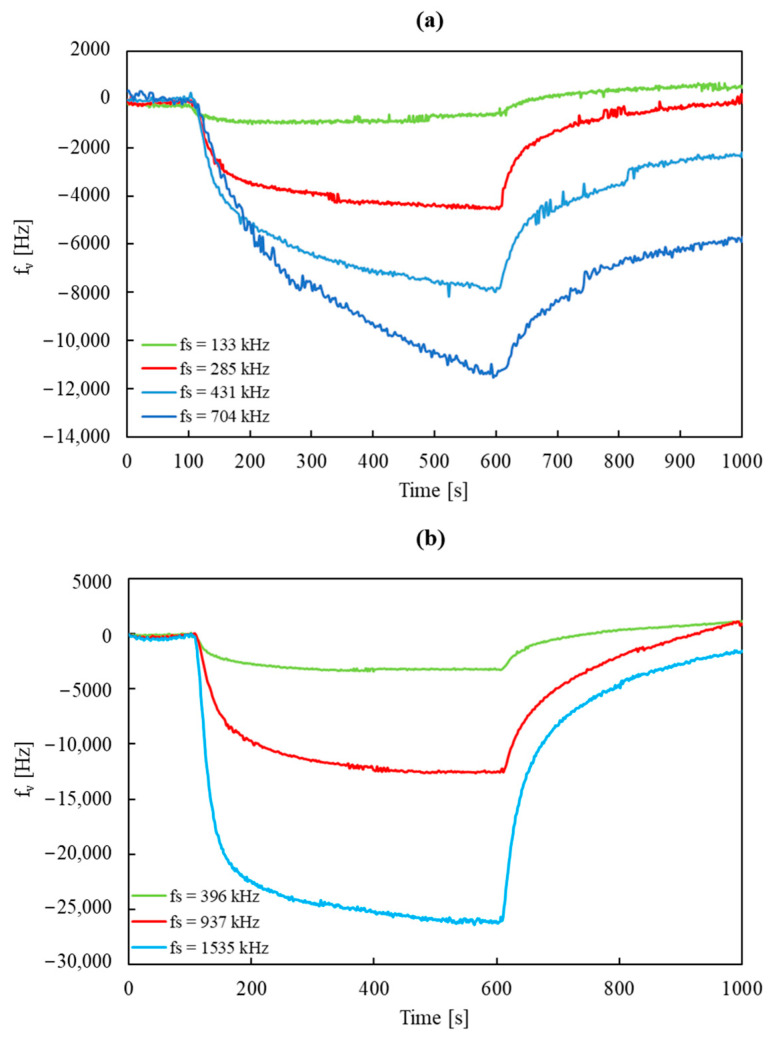
Plots of signal versus time for sensors using (**a**) R315 and (**b**) R433 resonators loaded with a polymer layer to varying degrees during exposure to DMMP vapor at a concentration of 3 mg/m^3^ (temperature approx. 25 °C).

Sensors with different resonance frequency shift values were tested in an atmosphere of DMMP vapor. These tests were carried out at a room temperature of approximately 25 °C during the measurement period (the measurement chamber was not thermostatically controlled). The sensors were exposed to a stream of dry air with a constant flow rate of 200 cm^3^/min and a DMMP concentration of 3 mg/m^3^ (0.54 ppm). During a single measurement lasting 1000 s, the sensor was subsequently exposed to dry carrier gas for 100 s, carrier gas with DMMP for 500 s, and dry carrier gas for 400 s. Figure 5 shows plots of signal versus time for sensors using R315 and R433 resonators loaded with a polymer layer to varying degrees.

Next, we sought to determine the metrological parameters of the sensors and compare the performances of the sensors using the R315 and R433 resonators. Table 2 lists the basic parameters for the analyzed sensors as follows: *f_s_*—shift of the resonance frequency resulting from the application of the polymer layer; *σ*—noise amplitude (equal to the standard deviation of the sensor signal baseline recorded during exposure to clean carrier gas for 100 s); *f_v_*—sensor signal for a gas with a DMMP concentration of 3 mg/m^3^ after 200 s from the beginning of exposure to this gas; and *σ*/*f_v_*—ratio of the noise amplitude to the signal amplitude proportional to the DMMP detection limit of the sensor.

The waveforms for the sensors using the R315 resonator (presented in Figure 5a) with layers causing a shift of the resonance frequency *f_s_* = 133, 285, and 431 kHz were characterized by good signal stability (noise values were similar, and their average was approximately 60 Hz). The waveform recorded for the fourth sensor (*f_s_* = 704 kHz) was characterized by much greater noise (noise amplitude was approximately 150 Hz). This may have been due to deterioration of the resonator’s *Q* factor caused by applying an excessively thick layer (this is visible in Figure 4a), resulting in a significant reduction in the amplitude and broadening of the resonance peak in the amplitude characteristic. Nevertheless, the absolute value of the signal amplitude recorded for this sensor was higher than in the case of the *f_s_* = 431 kHz sensor, these values being 7.94 and 6.43 kHz, respectively. To compare the usefulness of the R315 sensors characterized by the two thickest polymer layers, the ratios of the noise amplitude and the signal obtained 200 s after the beginning of exposure to DMMP were examined for both sensors. This ratio is proportional to the detection limit of the substance expressed according to IUPAC [20]:

LOD = 3σ/S,(1)

where σ represents noise amplitude and S represents sensitivity.

The value of this ratio for the *f_s_* = 431 kHz sensor was more than twice lower than that for the *f_s_* = 703 kHz sensor (8.99 and 18.69, respectively). On the basis of this finding, we concluded that the first of these sensors was able to detect lower concentrations of DMMP. This finding also indicated that the optimal polymer layer thickness for sensors using R315 resonators is less than 700 kHz.

Figure 5b presents the waveforms obtained for the R433 sensors with *f_s_* = 396, 937, and 1535 kHz. In all cases, noise amplitude was relatively small (compared to the signal amplitude), although noise increased with the increased thickness of the applied layer. Additionally, an *f_s_* = 2142 kHz sensor was prepared (the amplitude characteristics of this sensor are presented in Figure 4b), but due to a low *Q* value, the baseline signal of this sensor was very unstable, which made it impossible to carry out the measurement. On the basis of this finding, we concluded that the optimal polymer film thickness causes a shift in the resonance frequency of less than 2000 kHz.

Analyzing the values of the *σ*/*f_v_* ratio and assuming the limit values of the load of the R315 and R433 resonators (below which stable measurement is possible) to be approximately 700 and 2000 kHz, respectively, we may say that the R433 resonator is better suited for use in sensors. Considering the sensors with the lowest *σ*/*f_v_* ratio values manufactured using R315 (*f_s_* = 431 kHz, *σ*/*f_v_*·10^3^ = 8.99) and R433 (*f_s_* = 1535 kHz, *σ*/*f_v_*·10^3^ = 6.21), the latter exhibited significantly better results and would probably allow for the determination of lower DMMP concentrations. Here, it is also worth paying attention to the absolute value of the signal amplitude of this sensor, this being approximately 23.5 kHz. This value, at a DMMP concentration of 3 mg/m^3^, is one of the highest reported in the literature [21,22,23].

To compare our results with those obtained in other works on this topic, we reviewed the studies on sensor performance previously published in scientific journals. Because many factors affect sensor signal (relating both to measurement conditions and to the design of the transducer and the chemoselective coating) and these are not standardized across published works, we are aware that this comparison is of limited usefulness. However, to show the potential of the tested devices (R315 and R433) compared to other sensor transducers, we list the most important sensor parameters in Table 3. Please note that, in this table, we only include DMMP sensors that used polymer sensing layers, as in the present study.

The table above includes data for sensors based on resonators with a frequency of approximately 433 MHz (typically 433.92 MHz), among others. However, in some of these referenced works [28,29], it was not specified whether they were dedicated sensor devices. Additionally, in certain works [6,30], the sensors were based on resonators described as intended for radiocommunication applications (remote-control and wireless security). A comparison of our measurement results obtained using R315 and, especially, R433 with data from the literature shows that our sensors performed well. Considering the high signal values obtained for trace concentrations of DMMP along with the low cost and availability of these transducers, we believe they have great potential for use in gas sensors.

## 4. Conclusions

Two popular SAW resonators, R315 and R433, can be effectively used as transducers for gas sensors. The tests conducted on both of these resonators within their useful layer-loading range showed that the R433 performed even better in this role than its counterpart. Both devices are widely used in RF applications and are manufactured by many producers, making them an easily accessible and cost-effective alternative for dedicated transducers in gas-sensing applications.

It is also worth noting that, apart from the SAW resonators, the key element of our measurement system was an electronic reading system based on a budget-friendly, compact vector network analyzer, which is commonly used as a universal measurement device in radio communication. This shows that the detection of trace amounts of pollutants in gases is possible using a relatively simple device built with inexpensive and widely available components, typically used in other industrial applications.

## Figures and Tables

**Table 1:** Average basic parameters of resonators.

Type of Resonator	Unloaded *Q* Factor	50 Ω Loaded *Q* Factor	Insertion Loss [dB]	Temperature Stability [ppm/°C]	Static Capacitance [pF]
315 MHz	12,800	2000	1.5	0.037	2.7
433 MHz	12,000	1500	1.6	0.032	2.3

**Table 2:** Some metrological parameters of sensors using R315 and R433 resonators (explanation in the text).

SAW Device	*f_s_* [kHz]	*σ* [kHz]	*f_v_* [kHz]	*σ*/*fv*·10^3^
R315	133	0.05	1.01	50.48
	285	0.07	3.85	18.08
	431	0.06	6.43	8.99
	704	0.15	7.94	18.69
R433	396	0.05	3.20	15.28
	937	0.10	13.28	7.48
	1535	0.15	23.43	6.21
	2142 *	-	-	-

* Measurement failed due to unstable sensor signal.

**Table 3:** Selected key parameters of DMMP sensors using various SAW transducers and sensing layers made of hydrogen-bond acidic polymers reported in the literature.

Type of SAW Device/Center Freq. [MHz]	*f_s_* [kHz]	Temperature [°C]	DMMP Conc. [mg/m^3^]	Response [kHz]	Source
Delay line/158	250	30	1.1	1.3	[24]
Two-port resonator/195	100	30	1.5	0.62	[25]
Two-port resonator/300	170	20	1.5	4.0	[26]
Two-port resonator/195	100	30	1.5	0.5	[25]
Two-port resonator/417	500	Not available	0.1	2.2	[27]
Two-port resonator/433	770	15	1	3.2	[28]
Two-port resonator/433	740	15	1	10	[28]
Two-port resonator/433	1200	20	1	11	[29]
One-port SAW resonators/433	200	30	5.5	4.3	[6]
One-port resonator/433	600	25	28	35	[30]
One-port SAW resonators/315	700	25	3	7.9	This work
One-port SAW resonators/433	1500	25	3	23	This work

## Data Availability

The data presented in this study are available on request from the corresponding author.

## Supplementary Materials

The following supporting information can be downloaded at: https://www.mdpi.com/article/10.3390/s24134299/s1, Figure S1: Diagram of synthesis of 25–30% [3-(1,1,1,3,3,3-hexafluoropropan-2-ol)propyl]methylsiloxane dimethylsiloxane copolymer; Figure S2: FT-IR spectra of initial (25-30% methylhydrosiloxane) dimethylsiloxane copolymer hydride terminated (dotted line) and obtained 25–30% [3-(1,1,1,3,3,3-hexafluoropropan-2-ol)propyl]methylsiloxane dimethylsiloxane copolymer (solid line). IR spectra were recorded using the IR Tracer 100 device (Shimadzu, Japan) using the transmission technique (0.5 wt.% polymer solution in CCl4, ZnSe windows, optical path length 0.2 mm); Figure S3: Values of the resonance frequency shifts (fs) of the sensor using the R315 resonator obtained in a series of 10-fold application and washing of the sensing layer (points 2-11, polymer solution used: 0.5% by weight in THF). Point 1 means the bare resonator (after washing with solvents and glow discharge treatment), while point 12 is the fs value for the resonator from which the polymer layer was washed with clean solvents. The average fs value obtained using a 0.5% polymer solution is 422.3 kHz (S.D. 17.1 kHz); Figure S4: The fs values (blue) and amplitudes (red) of sensors based on the R315 resonator manufactured using polymer solutions of various percentage concentrations; Figure S5: Diagram of measuring system; Figure S6: DMMP diffusion source mass versus time plot.
