# Supplementary material for: The Application of Commercial Surface Acoustic Wave Radio Communication Filters as Transducers for DMMP Sensors
Source: Sensors (Basel). 2024 Jul 2;24(13):4299. doi: 10.3390/s24134299 (PMC11244062; doi:10.3390/s24134299)
Supplement: Supplementary file 1 [file sensors-24-04299-s001.zip › sensors-3061097-supplementary.pdf]

Supporting information for:

The Application of Commercial Surface Acoustic Wave Radio Communication Filters as Transducers for DMMP Sensors

Michał Grabka [1,*], Krzysztof Jasek [1], Mateusz Pasternak [2] and Zygfryd Witkiewicz [1]

[1] Faculty of Advanced Technologies and Chemistry, Military University of Technology, 00-908 Warsaw, Poland
[2] Faculty of Electronics, Military University of Technology, 00-908 Warsaw, Poland
* Correspondence: michal.grabka@wat.edu.pl

Contents:

1. Synthesis recipe of 25 – 30% [3-(1,1,1,3,3,3-hexafluoropropan-2-ol)propyl]methylsiloxane dimethylsiloxane copolymer.
2. Details of applying polymer layers to SAW devices.
3. Gas mixture generation system - methodology for determining the analyte concentration in the generated gas.

1. Synthesis recipe of 25 – 30% [3-(1,1,1,3,3,3-hexafluoropropan-2-ol)propyl]methylsiloxane dimethylsiloxane copolymer

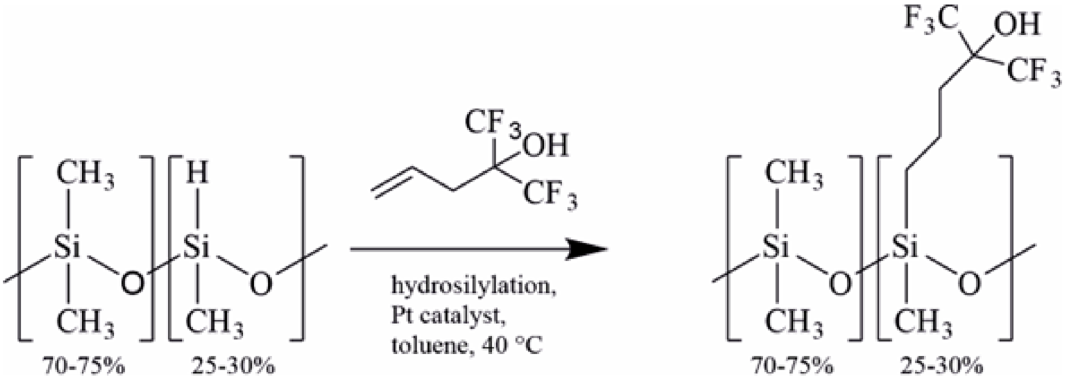

Figure S1. Diagram of synthesis of 25 – 30% [3-(1,1,1,3,3,3-hexafluoropropan-2-ol)propyl]methylsiloxane dimethylsiloxane copolymer.

The synthesis of the material consisted in modifying the chain of a copolymer of dimethylsiloxane and methylhydrosiloxane with a Si-H bond content of 25-30% ((25-30% methylhydrosiloxane) dimethylsiloxane copolymer hydride terminated, ABCR AB 146378, Germany) by introducing 2-allyl-1,1,1,3,3,3-hexafluoroisopropanol substituents (2-allylhexafluoroisopropanol, 98%, ABCR AB 134614, Germany) via a platinum catalyzed (Karstedt, platinum-divinyltetramethyldisiloxane complex in xylene, 2.1-2.4% Pt, ABCR AB 146697, Germany) hydrosilylation reaction. The reaction was carried out under N_2 with the

exclusion of moisture by use of Schlenk technique. To the mixture of substituent (0.009 mol) and Karstedt's catalyst (0.012 g) in dry toluene (10 cm^3) the dimethylsiloxane methylhydrosiloxane copolymer (1.5 g, 0.007 mol of Si-H – the amount of the polymer was calculated on the basis of the average content of monomers with Si-H bond adopted on the basis of the dimethylsiloxane methylhydrosiloxane copolymer manufacturer's declaration as 27.5%) was added slowly dropwise. The mixture was refluxed for 10 h in 40 ˚C under N_2. The progress of the reaction was monitored by FT-IR to observe the disappearance of the Si-H stretching band at 2157.6 cm^{-1}. After complete disappearance of this band mixture was cooled to room temperature. The mixture was then filtered under vacuum and the solvents removed by rotary evaporation. Finally, light grey oil was collected (2.67 g, yield: 79 %).

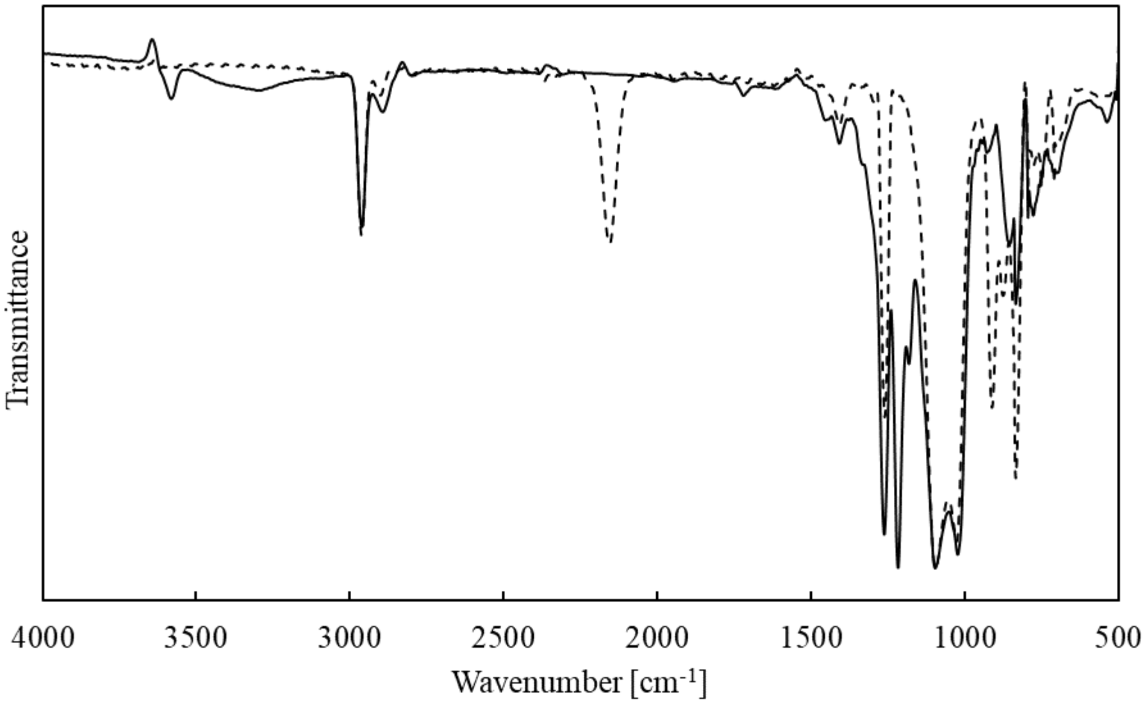

Figure S2. FT-IR spectra of initial (25-30% methylhydrosiloxane) dimethylsiloxane copolymer hydride terminated (dotted line) and obtained 25 – 30% [3-(1,1,1,3,3,3-hexafluoropropan-2-ol)propyl]methylsiloxane dimethylsiloxane copolymer (solid line). IR spectra were recorded using the IR Tracer 100 device (Shimadzu, Japan) using the transmission technique (0.5 wt.% polymer solution in CCl_4, ZnSe windows, optical path length 0.2 mm).

2. Details of applying polymer layers to SAW devices

The layers were applied by immersing the resonators in a polymer solution of various concentrations. After a few seconds, the resonator was removed from the solution and allowed to evaporate the solvent. Although this method was a significant simplification of classic dip

coating, in which, in addition to the concentration of the solution, other parameters are controlled, such as the speed of extraction from the liquid, it was possible to create layers with repeatable parameters. Figure 3s shows the values of the sensors' resonance frequency shifts (f_s) obtained when applying a polymer layer ten times by immersing the resonator in a solution with a polymer concentration of 0.5 %. Between application of subsequent layers, the resonator was washed using clean solvents (acetone, ethanol, THF).

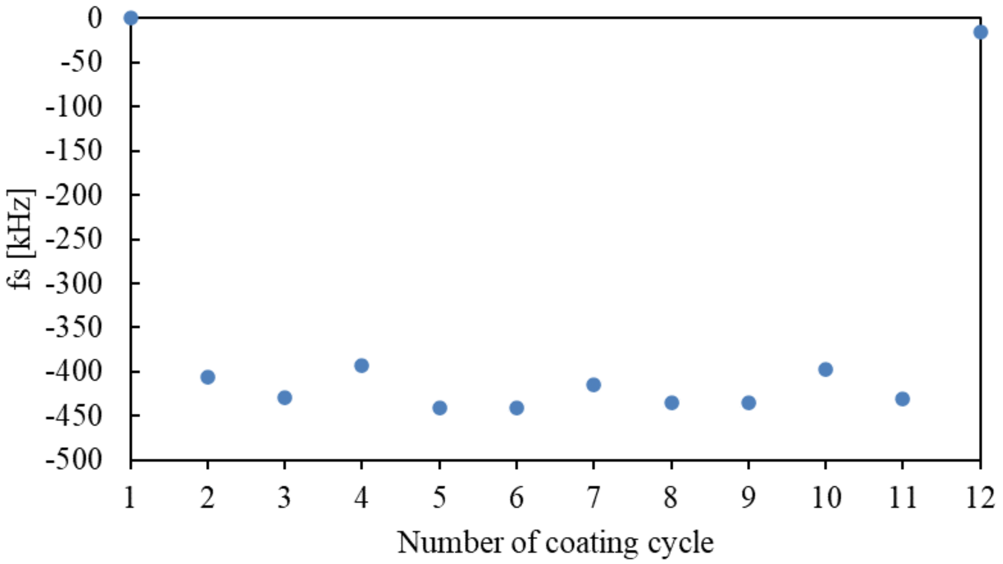

Figure S3. Values of the resonance frequency shifts (f_s) of the sensor using the R315 resonator obtained in a series of 10-fold application and washing of the sensing layer (points 2-11, polymer solution used: 0.5% by weight in THF). Point 1 means the bare resonator (after washing with solvents and glow discharge treatment), while point 12 is the f_s value for the resonator from which the polymer layer was washed with clean solvents. The average f_s value obtained using a 0.5% polymer solution is 422.3 kHz (S.D. 17.1 kHz).

By using polymer solutions of different concentrations, it was possible to apply polymer layers causing different shifts in the resonance frequency. The results obtained for three polymer solutions in THF with different polymer contents are presented below (Figure 4s).

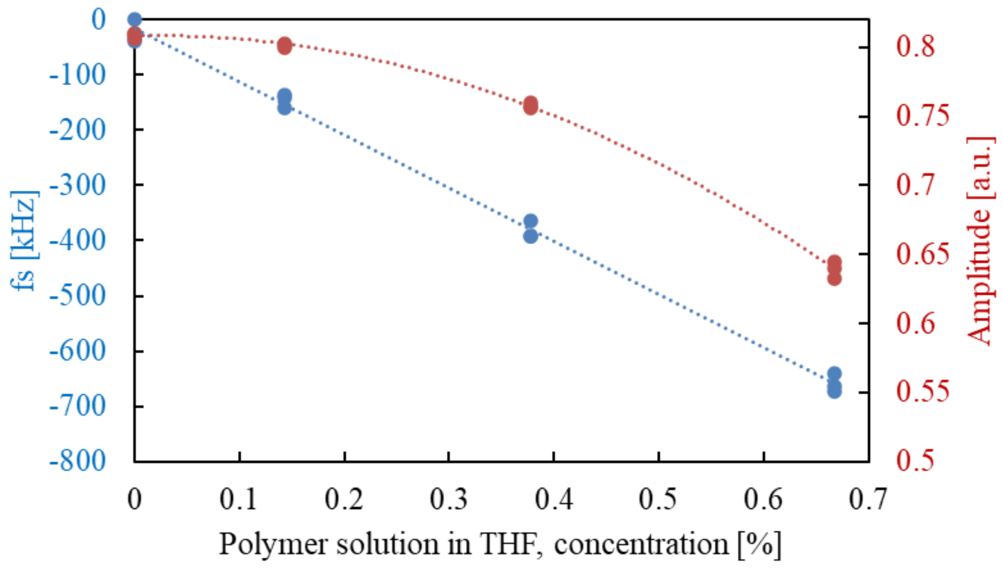

Figure S4. The f_s values (blue) and amplitudes (red) of sensors based on the R315 resonator manufactured using polymer solutions of various percentage concentrations.

The applied layers were stable for at least several weeks (between measurements, the sensors were stored in a chemical laboratory at a room temperature of approx. 25 °C and a relative air humidity of approx. 20%).

3. Gas mixture generation system - methodology for determining the analyte concentration in the generated gas.

The sensors were tested using a measuring system, the diagram of which is presented in the Figure 5s.

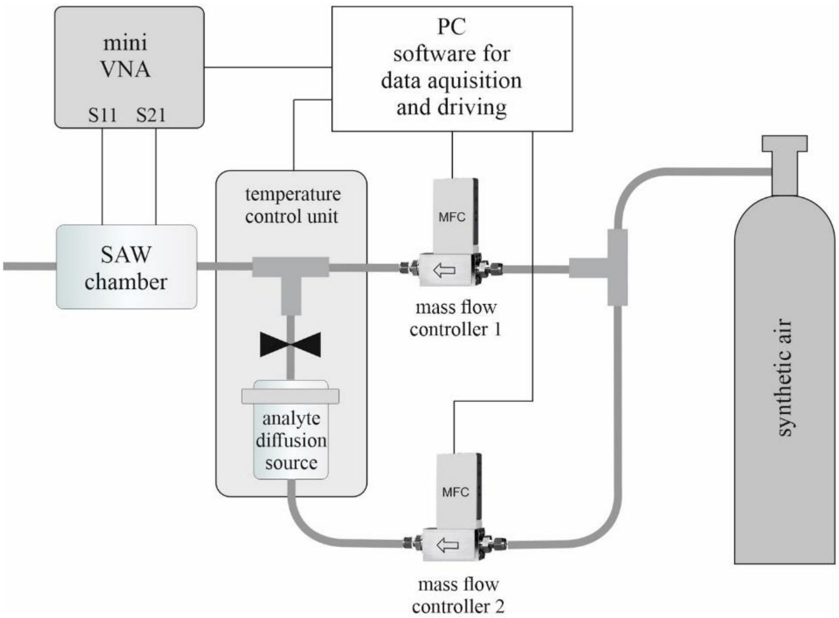

Figure S5. Diagram of measuring system.

Diffusion sources in the form of a small glass vessel (liquid analyte reservoir) ended with a capillary were used to generate specific concentrations of analyte in the stream of carrier gas. The phenomenon that determines a vapor emission from such a source is the diffusion of substance vapors through the gas layer filling the capillary. In practice, the emissions of each source have been determined empirically by gravimetric measurements. Prior and during the measurements, the source was stored at a constant temperature and periodically weighed on an analytical balance. The emission value for an analyte was calculated on the basis of the source mass loss Δm and the time between mass measurements Δt according to the relationship:

$$E = \frac{\Delta m}{\Delta t} \qquad (S1)$$

An example of the DMMP vapor source weighing results is shown in Figure 6s. Additionally, the calculated emission value (E_{DMMP}) is presented.

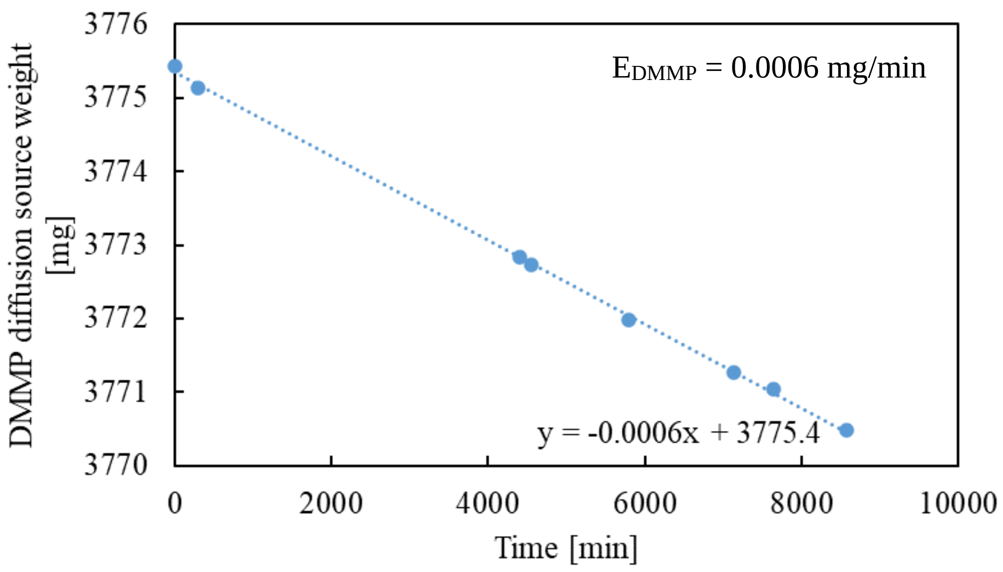

Figure S6. DMMP diffusion source mass versus time plot.

For constant emission, the concentration of the analyte in the gas mixture was controlled solely by changing the settings of the flow regulators q_1 and q_2. The equation for calculating the concentration value C [mg/m^3] is as follows:

$$C = \frac{E}{q_1 + q_2} \tag{S2}$$

where:

q_1 flow of dilution gas (mass flow controller 1),

q_2 flow of carrier gas through the container with sample diffusion source (mass flow controller 2).
